# Oviposition Substrate of the Mountain Fly *Drosophila nigrosparsa* (Diptera: Drosophilidae)

**DOI:** 10.1371/journal.pone.0165743

**Published:** 2016-10-27

**Authors:** Martin-Carl Kinzner, Magdalena Tratter, Gerhard Bächli, Martin Kirchmair, Rüdiger Kaufmann, Wolfgang Arthofer, Birgit C. Schlick-Steiner, Florian M. Steiner

**Affiliations:** 1 Institute of Ecology, University of Innsbruck, Innsbruck, Austria; 2 Institute of Evolutionary Biology and Environmental Studies, University of Zurich, Zurich, Switzerland; 3 Institute of Microbiology, University of Innsbruck, Innsbruck, Austria; Biomedical Sciences Research Center Alexander Fleming, GREECE

## Abstract

The survival of insect larvae often depends on the mother’s choice of oviposition substrate, and thus, this choice is an essential part of an insect species’ ecology. Especially species with narrow substrate preferences may suffer from changes in substrate availability triggered by, for example, climate change. Recent climate warming is affecting species directly (e.g., physiology) but also indirectly (e.g., biological interactions) leading to mismatching phenologies and distributions. However, the preferred oviposition substrate is still unknown for many drosophilid species, especially for those at higher elevations. In this study, we investigated the oviposition-substrate preference of the montane-alpine fly *Drosophila nigrosparsa* in rearing and multiple-choice experiments using natural substrates in the laboratory. Insect emergence from field-collected substrates was tested. More than 650 insects were reared from natural substrates, among them 152 drosophilids but no individual of *D*. *nigrosparsa*. In the multiple-choice experiments, *D*. *nigrosparsa* preferred ovipositing on mushrooms (> 93% of eggs); additionally, a few eggs were laid on berries but none on other substrates such as cow faeces, rotten plant material, and soil. The flies laid 24 times more eggs per day when mushrooms were included in the substrates than when they were excluded. We infer that *D*. *nigrosparsa* is a mushroom breeder with some variation in oviposition choice. The flies favoured some mushrooms over others, but they were not specialised on a single fungal taxon. Although it is unclear if and how climate change will affect *D*. *nigrosparsa*, our results indicate that this species will not be threatened by oviposition-substrate limitations in the near future because of the broad altitudinal distribution of the mushrooms considered here, even if the flies will have to shift upwards to withstand increasing temperatures.

## Introduction

Ovipositing on the preferred substrate can increase the likelihood of insect offspring’s survival [1], and especially poorly agile insect larvae strongly depend on the mother’s substrate selection. According to the major hypothesis on the evolution of oviposition choice, there should be strong selective pressure on oviposition substrate to enable optimum larval performance [2]. However, females often do not prefer the substrate optimising larval performance but rather their own performance [3,4]. This highlights that other factors than offspring survival may play a role in oviposition choice, such as host chemistry, predictability, genetics, and presence or absence of predators [5,6].

The fly family Drosophilidae is among the best studied animal taxa, and several drosophilid species are well characterised in, for example, physiology, genetics, life-cycle, developmental biology, and ecology [7,8]. The preferred oviposition substrate, however, is known for only a few drosophilids [9–11], and in-depth knowledge especially for species from higher elevations is lacking. Shorrocks [11] defined four categories of breeding substrates for European drosophilids, namely, decaying plant material, fermenting fruits, fungi, and sap fluxes of trees. Although many drosophilid species can use several feeding substrates as adults, the oviposition substrate preference is often narrower and not the same as the food preference [9,12]. Some species are highly specialised in their oviposition preference, such as *Drosophila pachea* [13]; others are more generalist breeders and are able to use various substrates for successful larval development, such as *D*. *subobscura* [14]. Furthermore, oviposition choice can vary within species [9,15].

One major factor of recent climate change is increasing temperature; worst-case scenarios predict increments of up to 4.8°C until the end of the 21^st^ century [16]. Species are affected directly, by physiological changes, and indirectly, by effects on interacting species [17]. Distribution ranges and synchrony of interacting partners can become mismatched so that the overlapping area and period are reduced, respectively [18,19]. Models predict increasing mismatches, spatially and temporally [20], which can result in the extinction of interacting species [21]. Insect species with narrow substrate preferences for egg laying are especially vulnerable [17]. Hence, knowledge of the preferred oviposition substrate is a crucial factor when predicting a species’ future under changed environmental conditions such as under climate warming.

*Drosophila* (*Drosophila*) *nigrosparsa* Strobl, 1898 is distributed in European mountain regions, where it is most abundant at the timber line [22]. Currently, we are establishing *D*. *nigrosparsa* as a new study system for climate change research (Austrian Science Fund, project number P 23949, https://pf.fwf.ac.at/project_pdfs/pdf_abstracts/p23949e.pdf). This species was chosen because the genus *Drosophila* contains some of the best studied animals [7], and among drosophilids, only *D*. *nigrosparsa* is both confined to high altitudes and culturable in the laboratory. In this project, we are performing laboratory selection experiments to investigate the ability of this fly species to adapt to increasing temperatures in the alpine ecosystem. The species’ transcriptome has been sequenced [23], and various life history traits and physiological limits of *D*. *nigrosparsa* have been characterised under laboratory conditions (P. Krapf, M.-C. Kinzner, M. Nindl, C. Heussler, A. A. Hoffmann, W. Arthofer, B. C. Schlick-Steiner, F. M. Steiner, unpubl.). However, there is currently no information available concerning the oviposition preference of *D*. *nigrosparsa*. This can hamper progress in climate change research—even if the fly is able to adapt to higher temperatures, an interacting partner might not be. To illuminate this: Assume that *D*. *nigrosparsa* is specialised to oviposit on species X, both having a similar altitudinal distribution at the timberline in the Alps. Then, if temperatures in the Alps rise strongly until the end of the 21^st^ century, as predicted [24], *D*. *nigrosparsa* might not be able to adapt to the higher temperatures, but species X might be. In this situation, *D*. *nigrosparsa* would migrate to higher altitudes, but species X would keep its recent distribution, as observed for other biotic observations (e.g., [19,20]), leading to an increasing distribution mismatch. Once the two species’ distributions do not overlap any more, *D*. *nigrosparsa* would lose its biological partner, and, due to the fly’s specialised oviposition biology, at least some of its populations might vanish. This is just one theoretically possible future scenario, but it stresses the importance for climate change research to gain information about biological interactions in addition to selection experiments in the laboratory.

In the following, we addressed three questions: (i) What natural substrate is preferred by *D*. *nigrosparsa* for oviposition; (ii) is *D*. *nigrosparsa* a generalist or a specialist concerning oviposition substrate; and (iii) is *D*. *nigrosparsa* opportunistic when the preferred substrate is not available, changing oviposition to non-preferred substrates?

## Materials and Methods

### Origin and maintenance of experimental flies

In summer 2010, *Drosophila nigrosparsa* was collected using fermented-banana baits at Kaserstattalm (Tyrol, Austria, 11.29°E 47.13°N) at 2000 m above sea level. No specific permissions were required because none of the species used in this study is endangered or protected. Flies were kept in the laboratory as mass bred population using grape agar medium (30 g agar, 1000 mL deionised water, 334 mL grape juice, 3.4 g methyl-4-hydroxybenzoate, and 34 g sucrose; modified from Sullivan et al. [25]) and malt medium (10 g agar, 1000 mL deionised water, 15 g dried yeast, 100 g malt, 3 g methyl-4-hydroxybenzoate, 3.6 mL propionic acid, and 50 g semolina; modified from Lakovaara [26]) at 19°C and ca. 60% relative humidity in a 16L:8D photoperiod. After about 100 days, a pair of flies was isolated for oviposition on grape agar to initiate a strongly inbred laboratory population. After establishment, laboratory populations were maintained at a population size of approximately 200 for four years.

### Experiment 1—Rearing experiment

In August 2012, 239 substrates (S1 Table) were collected at three natural habitats of *D*. *nigrosparsa* at 2000 m above sea level, at Arztal (Tyrol, Austria, 11.50°E 47.18°N), Kaserstattalm, and Pfitscherjoch (South Tyrol, Italy, 11.68°E 46.98°N), and brought to an environmental chamber (DWM, Weiss Technik, Germany; modified) at the University of Innsbruck. Temperature and relative humidity in the chamber were 19°C and 50–70%, respectively, in a 16L:8D photoperiod. The surface of substrates was disinfected using a mixture of 1000 mL deionised water, 3 g methyl-4-hydroxybenzoate, and 3.6 mL propionic acid (pH 8.9). Substrates were placed individually in 500 mL plastic cups containing ca. 40 mL malt medium. Emerged adults were collected weekly and stored in 96% ethanol at -20°C. After three months, the chamber was cooled down to 1–4°C to simulate winter. Five weeks later, the chamber was warmed up again to 19°C, and substrates were controlled for emerged adults for another two months. *Drosophila* species were identified morphologically according to Bächli & Burla [22].

### Experiment 2—General oviposition preference

**Fig 1:**
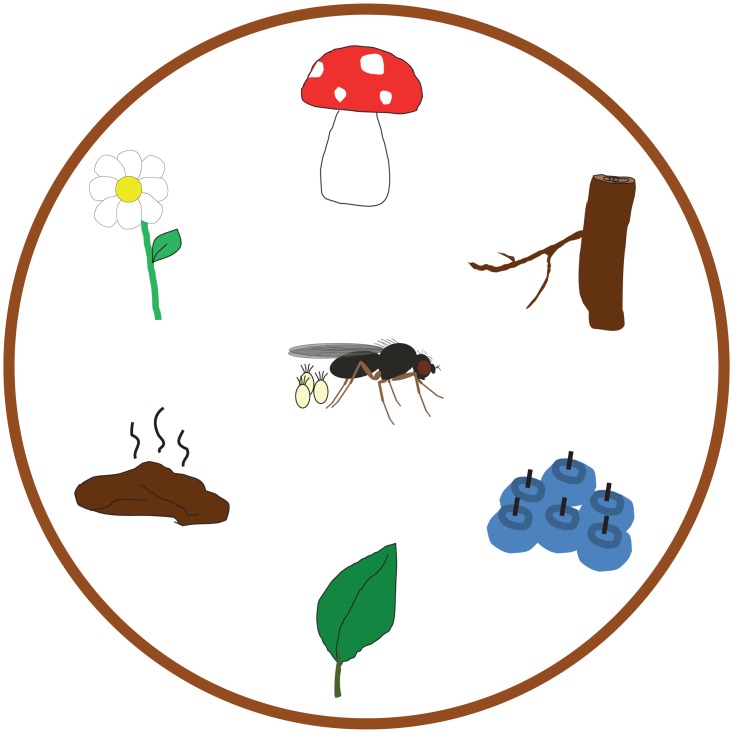
Schematic illustration of the multiple-choice oviposition Experiments 2–4. The brown circle indicates the cage. Size relations do not represent the real situation.

In September 2014, 24 substrates were collected at Kaserstattalm (S2 Table) and brought to the environmental chamber used also in Experiment 1. Temperature, relative humidity, and photoperiod in the chamber were equal to Experiment 1. Substrates were placed individually in 90-mm-diameter Petri dishes, which were randomly positioned along the inner side of a circular untransparent plastic cage of 530 mm diameter with a transparent observation window at the top (Fig 1). For water supply, a Petri dish containing ca. 40 mL 3.0% plain agar was placed in the centre of the cage. Seventy females and 40 males of mixed age of the inbred laboratory population were briefly CO_2_ anaesthetised for sexing and released on the plain agar equidistantly from the 24 potential oviposition substrates. After 54 hours, the number of eggs laid on each substrate was counted under the microscope (Nikon SMZ-10A, Nikon Corporation, Japan, 7.5 − 49x magnification). Three replicate cages were run in parallel. To control for potentially overlooked eggs after recording the number of eggs, substrates on which no eggs had been detected were placed individually in 500 mL plastic cups containing 8 mL malt medium enabling the development of eggs to adulthood. Emerged adults were collected weekly for 14 weeks.

### Experiment 3—Generalist vs. specialist

In September 2014, additional samples of blueberries (fruits of *Vaccinium myrtillus*), bog bilberries (fruits of *V*. *uliginosum*), and fruiting bodies of seven different fungi (all species detectable at that time) were collected at Kaserstattalm (S3 Table) and brought to the environmental chamber used also in Experiments 1 & 2. Substrates were placed individually in 55-mm-diameter Petri dishes containing ca. 10 mL 0.4% plain agar to prevent desiccation. As mushrooms were determined morphologically after the experiment [27] using photographs, one dish inadvertently contained two different species (*Lycoperdon* sp. & *Bovista* sp.). Dishes were randomly positioned along the inner side of a circular untransparent plastic cage of 270 mm diameter with a transparent observation window at the top (Fig 1). A Petri dish containing ca. 10 mL 3.0% plain agar was added as negative control. For water supply, a 90-mm-diameter Petri dish containing ca. 40 mL 3.0% plain agar was placed in the centre of the cage. Fifty females and 30 males of mixed age of the inbred laboratory population were briefly CO_2_ anaesthetised for sexing and released on the plain agar. The experimental procedure was equal to that in Experiment 2, but the time for egg laying was extended to 64 hours.

### Experiment 4—Opportunistic ovipositing

In September 2014, nine not-favoured substrates from Experiment 2 (S4 Table) were recollected at Kaserstattalm based on a subjective choice of those substrates on which, according to the main oviposition types [11] and to personal observations, *D*. *nigrosparsa* might possibly lay eggs in the absence of mushrooms. The experimental design was the same as in Experiment 3.

Lacking knowledge about oviposition behaviour and clutch sizes, there was no justifiable specific probability model for the egg numbers. Therefore, we took a pragmatic approach and Box-Cox transformed (after adding 1 to handle zeroes) the egg counts to meet the criteria for an analysis of variance (ANOVA). Substrates without any eggs over all replicates having zero variance were excluded from analysis. The transformed data were analysed using ANOVA with Bonferroni-corrected post-hoc tests to compare substrates in R version 3.1.1 [28]. For homogenous groups shown in graphs, a p-level of 0.05 was applied.

### Molecular analysis

DNA of four drosophilids eclosed from Experiment 1 and of 39 *D*. *nigrosparsa* adults eclosed from Experiments 2–4 was extracted using the GenElute Mammalian Genomic DNA miniprep kit (Sigma, USA). PCR amplification of a mitochondrial COI gene stretch for species identification of drosophilids eclosed in Experiment 1 was carried out in a 10 μL reaction volume with 1.0 μL template DNA, 1× Rotor-Gene Probe PCR Master Mix (Qiagen, Germany), and 0.2 μM forward and reverse primers (UEA5 and UEA10, respectively; [29]). PCRs were performed at 94°C for 2 min followed by 35 cycles at 94°C for 30 s, 50°C for 45 s, 72°C for 2 min and a final extension at 72°C for 10 min.

We were aware that by counting eggs under the microscope, some eggs could have been overlooked (e.g., because of deep insertion in substrates), and some eggs (and/or larvae, pupae) could have been already on substrates before bringing them to the laboratory. Thus, the population of origin (wildtype or inbred laboratory population) of adults emerged from substrates on which no eggs were found in Experiments 2–4 (S2–S5 Tables) were identified using molecular markers. Therefore, flies were genotyped using five polymorphic microsatellite loci, namely, DN37, DN40, DN41, DN48, and DN49 [30]. PCR for genotyping was performed in a 5 μL reaction volume with 0.5 μL template DNA, 1× reaction buffer (Bioline, UK), 0.2 μM fluorescent-labelled M13 primer, 0.02 μM M13 tailed locus specific forward primer, 0.2 μM untailed specific reverse primer, and 0.25 U MyTaq polymerase (Bioline). For loci DN37, DN41, DN48, and DN49, cycling conditions were 94°C for 2 min followed by 35 cycles at 94°C for 30 s, 55°C for 45 s, 72°C for 1 min and a final extension at 72°C for 10 min. For locus DN40, cycling conditions were equal but annealing temperature was 48°C. Fragment analysis was performed on an ABI 3130 genetic analyzer (Applied Biosystems, USA). Traces were visualised and scored manually using PeakScanner Software v2.0 (Applied Biosystems). GeneClass v2.0 [31] was used for the classification of the population of origin (wildtype or inbred laboratory population) by using a two-step approach: First, the software was trained with microsatellite data from the wildtype and the inbred laboratory population already available [30] using the Bayesian method of Rannala & Mountain [32]. Second, as more than ten generations of laboratory evolution occurred between the inbred laboratory population characterised in 2013 [30] and the flies used in this study, all eclosed individuals from this study unambiguously assigned to the inbred laboratory population in the first step were used to train the software again. Individuals not assigned unambiguously to the inbred laboratory population in the first step were classified again using the new training set.

## Results

### Experiment 1—Rearing experiment

A total of 614 adult Diptera from 14 families were reared from 60 different substrates (S6 Table) whereby most individuals emerged from cow and sheep faeces and from mushrooms. The only drosophilid species was *Drosophila transversa* (GenBank accession numbers KU934279–KU934282), with all 152 individuals eclosing from mushrooms. In addition to dipterans, 11 Coleoptera, 20 Hemiptera, 2 Hymenoptera, and 41 Thysanoptera emerged (S6 Table).

### Experiment 2—General oviposition preference

**Fig 2:**
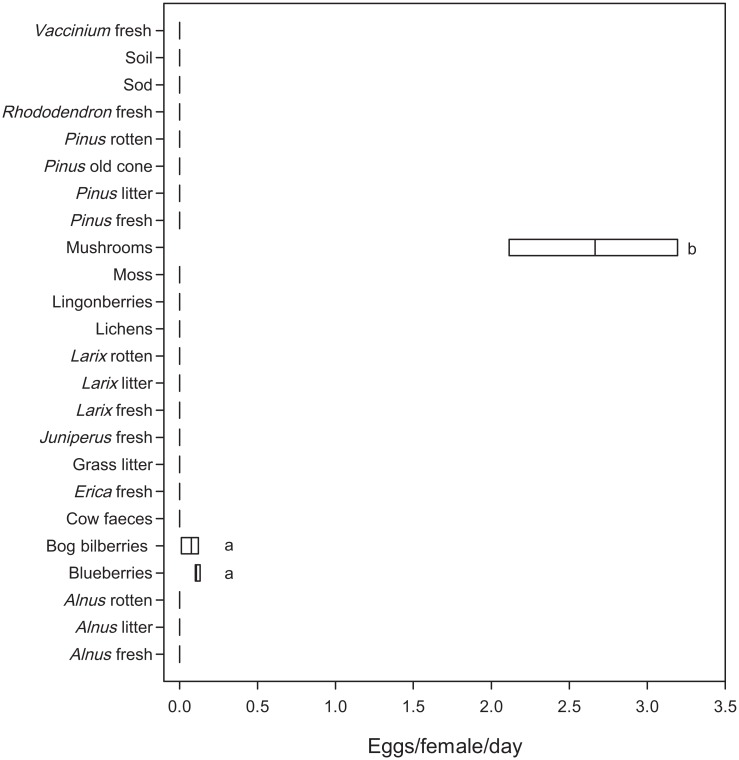
Number of eggs/female/day laid on various substrates from Experiment 2—General oviposition preference. The same lower case letters indicate homogenous significance groups (p = 0.05). For details about substrate nomenclature, see S1 Table.

Females of *D*. *nigrosparsa* oviposited on only three of the 24 offered substrates (Fig 2, S2 Table), consistently across all replicates. A total of 1343 eggs (2.842 eggs/female/day) were laid across all replicates. Most eggs were laid on the mixed mushroom sample, on average 2.658 ± 0.540 eggs/female/day (93.5 ± 19.0% of all eggs in Experiment 2, mean ± standard deviation). Only a few eggs were laid on blueberries and bog bilberries, on average 0.114 ± 0.017 (4.0 ± 0.6%) and 0.070 ± 0.054 eggs/female/day (2.5 ± 1.9%), respectively. The number of eggs differed significantly among substrates (Fig 2, Table 1). Blueberries and bog bilberries were not different in the pairwise comparison, but both differed significantly from mushrooms (S7 Table). After placing the substrates on malt medium, adults emerged from the three substrates on which eggs had been detected but from no other substrate (S2 Table).

**Table 1:** Results of the analysis of variance for Experiments 2–4.

	Factor	Df	SS	MS	F-value	p-value
Experiment 2	substrate	2	748.67	374.33	109.63	1.89 E^-5^
	error	6	20.49	3.41		
Experiment 3	substrate	8	335.11	41.89	13.00	4.75 E^-6^
	error	18	57.98	3.22		
Experiment 4	substrate	2	1.37	0.69	0.89	0.46
	error	6	4.61	0.77		

Df, degrees of freedom. SS, sum of squares. MS, mean square.

### Experiment 3—Generalist vs. specialist

**Fig 3:**
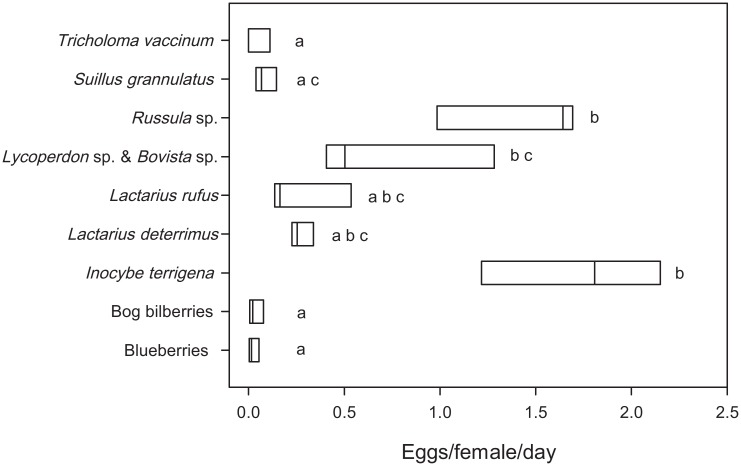
Number of eggs/female/day laid on various substrates from Experiment 3—Generalist vs. specialist. The same lower case letters indicate homogenous significance groups (p = 0.05).

Eggs were laid on all substrates at least in one replicate (Fig 3, S3 Table). In total, 1873 eggs (4.683 eggs/female/day) were counted across all replicates. The most attractive substrate was the mushroom *Inocybe terrigena* (36.3 ± 13.4% of all eggs in Experiment 3, mean ± standard deviation), followed by *Russula* sp. (29.3 ± 11.3%) and *Lycoperdon* sp. & *Bovista* sp. (17.3 ± 13.7%). The least attractive were blueberries (0.6 ± 0.8%), bog bilberries (0.9 ± 1.1%), and the mushroom *Tricholoma vaccinum* (1.1 ± 1.9%). The number of eggs differed significantly among substrates (Fig 3, Table 1); three homogenous substrate groups were found in post-hoc tests (S7 Table). In the sample *Lycoperdon* sp. & *Bovista* sp., only one egg was laid on *Bovista* sp. (replicate B); all other eggs were found on *Lycoperdon* sp. Adults emerged from two of four substrates (blueberries, bog bilberries, S3 Table) where zero eggs were counted; all had inbred laboratory population genotypes (S5 and S8 Tables).

### Experiment 4—Opportunistic ovipositing

**Fig 4:**
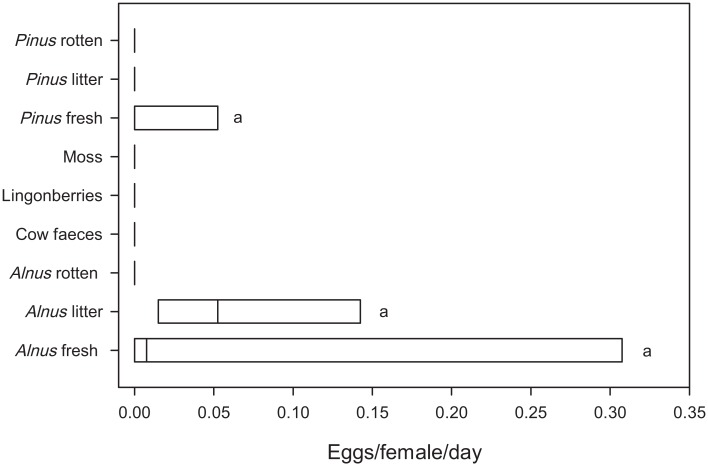
Number of eggs/female/day laid on various substrates from Experiment 4—Opportunistic ovipositing. The same lower case letters indicate homogenous significance groups (p = 0.05). For details about substrate nomenclature, see S1 Table.

A total of 77 eggs (0.193 eggs/female/day) were laid across all replicates (Fig 4, S4 Table), whereof 70 (90.9%) were found on fresh *Alnus* or *Alnus* litter. The remaining seven eggs (9.1%) were laid on fresh *Pinus* (replicate B). The number of eggs did not differ significantly among substrates in the ANOVA (Fig 4, Table 1) and in the post hoc tests (S7 Table). In addition, adults emerged from five of 21 substrates (*Alnus* fresh, cow faeces, lingonberries, *Pinus* fresh, and *Pinus* rotten, S4 Table) where no eggs had been detected. All had inbred laboratory population genotypes (S5 and S8 Tables).

## Discussion

### What natural substrate is preferred by *D*. *nigrosparsa* for oviposition?

Information on the oviposition preference is scarce for many insects, but oviposition substrate selection is an essential part of a species’ ecology [12]. We reveal here that the mountain fly *Drosophila nigrosparsa* prefers ovipositing on fungal fruiting bodies (Fig 2, S2 Table). Including this fly, 15 drosophilid species are now known to oviposit on fungi in Europe, of which 14 are *Drosophila* and one *Scaptomyza* [33]. Burla & Bächli [34] and Burla et al. [14] reared 11 *Drosophila* species from mushrooms in Switzerland lacking, apart from *D*. *nigrosparsa*, also *D*. *transversa* and *D*. *subobscura*, which both oviposit on fungi in England [35]. The latter two species are common at the natural habitat at Kaserstattalm, and thus, competition among them and *D*. *nigrosparsa* is possible by sharing the same resource, a topic now open for future research.

The rearing experiment in this study (Experiment 1) yielded 614 Diptera, among them 152 Drosophilidae (Table 1) but no *Drosophila nigrosparsa*. The only drosophilid species found was *D*. *transversa*, which was also reared from fungi in England [35] but was missing in a study in Switzerland, probably because of low elevation and latitude [14]. However, after knowing the mushroom preference of *D*. *nigrosparsa*, we would have expected at least a small rearing success from fungi. Probably, our failure reflects the low frequency of *D*. *nigrosparsa* in nature [22] in combination with the “aggregation model of species coexistence” [36,37], which assumes that females gregariously oviposit on resource patches, so that some patches are strongly occupied and a large number of patches remain uninhabited. By chance, we may not have collected occupied patches in our experiment. Possibly, not all substrates indeed relevant for *D*. *nigrosparsa* were considered here due to experimental limitations and seasonal variation in substrate availability. As we have searched intensively for any oviposition substrate mentioned in the literature, however, we are positive that at least various types of each substrate category according to Shorrocks [11] were included. For a final confirmation of our assumptions concerning *D*. *nigrosparsa*, we suggest additional field experiments and long-term studies focusing on changing distributions of both the flies and the mushrooms as well as on possible adaptations to rising temperature and other environmental factors.

### Is *D*. *nigrosparsa* a generalist or a specialist concerning oviposition substrate?

Species specialised on a single food or habitat source are predicted to be more threatened by changing environments than generalists [17]. Thus, information on the degree of specialisation helps making more reliable predictions of a species’ future survival. Even though *D*. *nigrosparsa* prefers mushrooms for oviposition (Fig 2), and some mushrooms were more attractive than others (Fig 3), the fly seems not to be specialised on a single fungal species or genus. Moreover, we think that the variation among replicates in Experiment 3 corroborates this conclusion. This finding is in accordance with those for most other drosophilid species of the fungus guild, which was explained by the unpredictability of many fungal fruiting bodies as the fruiting mainly depends on temperature and rainfall [38]. However, at least some fruiting bodies of various mushroom species can be found during the whole vegetation period also in high altitudes, although species richness decreases with increasing elevation [39]. Correlated with increasing temperature, the number of fungus species as well as the number of fungal fruiting bodies increased over the past decades [40]. Moreover, most fungi were observed to prolong their fruiting period in spring and autumn [41]. Hence, mushrooms will most probably not be a limiting resource in the future, even at higher altitudes. Currently, we are performing laboratory selection experiments to assess the evolutionary potential of *D*. *nigrosparsa* to adapt to increased temperature. Although we do not know if and how *D*. *nigrosparsa* will be affected by climate change, it seems unlikely that the oviposition substrate will be a limiting resource for *D*. *nigrosparsa* (and other fungus breeders) in the near future, even not under scenarios of the flies migrating vertically to habitats with more adequate temperatures. However, on the longer run, when temperatures will have risen even further, additional factors such as soil formation in high altitudes [42] may play an important role in the limitation of fungi and fungus-associated species.

### Is *D*. *nigrosparsa* opportunistic when the preferred substrate is not available, changing oviposition to non-preferred substrates?

Mismatches among interacting species are predicted to increase with ongoing climate change [20]. Thus, it might become advantageous, or even unavoidable, to oviposit opportunistically on others than the preferred substrate in the future. However, in our study, *D*. *nigrosparsa* reduced oviposition by 96% when the preferred substrate was not available: 0.193 eggs/female/day were laid on non-favoured substrate (Fig 4) versus 4.683 eggs/female/day on favoured substrates (Fig 3). Although there was some variation among replicates, this does not invalidate the conclusion of reduced oviposition behaviour—these results have to be seen relative to the oviposition behaviour in experiments with mushrooms present (Experiment 2 & 3), in which the flies laid up to 24 times more eggs. Even the maximum number of eggs laid in Experiment 4 was more than four times lower than the mean number of eggs on mushrooms in Experiment 2. Moreover, even on the laboratory substrate they had become accustomed to over four years (grape agar with malt and yeast, see Materials and Methods for details), flies from the same lines and generation laid fewer eggs than on mushrooms (2.760 eggs/50 females/day, data not shown). After knowing this strong preference for mushrooms, an optimisation of the laboratory media would potentially increase the flies’ reproductive success for further culturing. Although the flies laid a few eggs on blueberries and bog bilberries (Figs 2 & 3), fruits are unlikely to be the major oviposition substrate and likely are an alternative for egg laying just because of the late and short fruiting period of berries at high altitudes. In contrast, mushrooms are available during the whole vegetation period. The oviposition on sap fluxes and decaying plant material, which are also possible oviposition substrates of drosophilids in Europe [11], was avoided by *D*. *nigrosparsa* in our experiments, except when the favoured substrates were not offered (Fig 4). We conclude that *D*. *nigrosparsa* generally does not perform opportunistic ovipositing, but that there is some variation in oviposition choice.

In a few of our samples of Experiments 3 & 4, adults emerged where no eggs had been observed visually (S3–S5 Tables). Thus, egg-counting seems not to be an error-free method but a simple proxy for oviposition preferences. Probably, eggs had been overlooked because of deep insertion in the substrate, undetectable by examining the substrates’ surface, and possibly, those substrates had contained eggs already laid by wildtype females in nature. It would have been an important finding if eclosed adults had been of the wildtype genotype as this would have proven the substrate under question as oviposition substrate in nature. However, all eclosed adults had inbred laboratory population genotypes lacking wildtype-specific alleles (S5 Table). Thus, we infer that these eggs were laid by inbred laboratory population females and had not been introduced from the field. We also note that the lacking proof of the concerned substrates as oviposition substrates in nature does not bear on our laboratory-based inferences, given the low density of *D*. *nigrosparsa* in the field [22].

When putting a group of flies together (70 females in Experiment 2 and 50 females in Experiment 3 & 4), the oviposition behaviour of single females might not be independent from the behaviour of other individuals. Preliminary experiments with *D*. *nigrosparsa* showed that when individualising pairs of flies, oviposition ceased nearly completely. This observation is in line with gregarious oviposition behaviour assumed in nature [43]. Thus, performing the experiments with single individuals was not possible.

In future experiments, a higher number of replicates would be preferable to increase statistical power. Anyway, four reasons limit the number of feasible replicates: First, in the Alps, some of the relevant substrates are available for just a short period, such as blueberries and lingonberries (August to September, depending on the weather during the vegetation period). Second, *D*. *nigrosparsa* has a low oviposition rate and a low egg-to-adult survival rate (4 eggs/female/day and 20%, respectively, data not shown), at least in the laboratory. Thus, the number of adult flies available is limited, and for the presented experiments, we have used as many flies as possible at that time. Third, *D*. *nigrosparsa* has a relatively long minimum egg-to-adult developmental time (60 days, data not shown) compared with, for example, *D*. *melanogaster* (9 days [8]). This is an additional limiting factor for the number of flies available and for the synchronisation with various substrates. Fourth, climate chambers are limited in size. For true replicates, it is necessary to have all replicates in parallel in the same chamber; otherwise, the results could be influenced by different conditions in different chambers or in the same chamber at different points in time. Here, we placed as many replicates in parallel as possible in the available climate chamber. We do not believe that increasing the number of replicates would have changed the conclusions of our study, in that the major results were congruent among replicates. Anyway, validating our findings by larger-scale investigations will be desirable.

## Conclusion and Outlook

We showed that *Drosophila nigrosparsa* prefers ovipositing on fungal fruit bodies. This fly species seems to have preferences among mushrooms for oviposition, but is not specialised on a single fungal taxon. Opportunistic ovipositing on not-favoured substrates, that is, others than mushrooms, is generally not performed, but not impossible, highlighting some variation in oviposition choice. Due to the broad altitudinal distribution of the fungi considered here and *D*. *nigrosparsa*’s generalist ovipositing among mushrooms, we assume that the oviposition substrate will not limit *D*. *nigrosparsa* in the near future, even if the species will have to migrate vertically when temperatures are rising.

By being mostly descriptive, studies on basic life-history traits are currently not in vogue [44,45]. Nevertheless, information on life-history traits is highly important for a deeper understanding of a species’ ecology and evolution [46–49] and is available only for a few organisms [50]. We suggest—in addition to studies on the molecular baseline, not instead of them—focussing research on basic life-history traits to gain a more complete picture of a species’ biology.

## Supporting Information

S1 Table Substrates collected at the natural habitat of *Drosophila nigrosparsa* for Experiment 1—Rearing experiment. (DOC)

S2 Table Experiment 2—General oviposition preference. (DOC)

S3 Table Experiment 3—Generalist vs. specialist. (DOC)

S4 Table Experiment 4—Opportunistic ovipositing. (DOC)

S5 Table GeneClass assignment results of genotypes of individuals eclosed from substrates where no eggs had been found using five microsatellite loci. (DOC)

S6 Table Identification of eclosed adults from Experiment 1—Rearing experiment. (DOC)

S7 Table Significance matrix of the Bonferroni-corrected post-hoc tests for egg counts of Experiment 2–4. Significant comparisons (p = 0.05) are marked in red. For details about substrate nomenclature, see S1 Table.
(XLS)

S8 Table Genotypes for five polymorphic microsatellite loci (DN37, 40, 41, 48, and 49) of each individual used for GeneClass analysis of Experiments 3 and 4. Wildtype and inbred laboratory population strains (KG0L0 & ILP) were used as references for the classification of emerged adults.
(XLSX)
