# Supplementary material for: Oviposition Substrate of the Mountain Fly Drosophila nigrosparsa (Diptera: Drosophilidae)
Source: PLoS One. 2016 Oct 27;11(10):e0165743. doi: 10.1371/journal.pone.0165743 (PMC5082818; doi:10.1371/journal.pone.0165743)
Supplement: S1 Table — (DOC) [file pone.0165743.s001.doc]

| **ID** | **Substrate** |
| --- | --- |
| A1 | Lichens on *Picea abies* branch |
| A2 | *Alnus viridis* fresh |
| A3 | *Hieracium hoppeanum* |
| A4 | Lichens on deadwood |
| A5 | *Alnus viridis* litter and soil |
| A6 | Moss and soil |
| A7 | Moss and soil |
| A8 | Moss and soil |
| A9 | Dried mushroom indet. |
| A10 | *Vaccinium uliginosum* fruits (bog bilberries) |
| A11 | Lichens and soil |
| A12 | Lichens and soil |
| A13 | *Erica* fresh |
| A14 | Mushroom indet. |
| A15 | *Sempervivum arachnoideum* and soil |
| A16 | *Rhododendron* fresh |
| A17 | *Rhododendron* fresh |
| A18 | Moss and soil |
| A19 | Moss and soil |
| A20 | *Knautia dipsacifolia* |
| A21 | *Alnus incana* fresh with fruits |
| A22 | *Cirsium heterophyllum* |
| A23 | *Vaccinium vitis-idea* fruits (lingonberries) |
| A24 | Deadwood |
| A25 | *Cirsium heterophyllum* |
| A26 | *Juniperus* litter |
| A27 | *Juniperus* litter |
| A28 | *Erica* fresh and soil |
| A29 | Moss and soil |
| A30 | *Alnus viridis* litter |
| A31 | *Alnus viridis* litter |
| A32 | Mushroom indet. |
| A33 | Moss and soil |
| A34 | Moss and soil |
| A35 | Moss |
| A36 | Moss |
| A37 | *Alnus viridis* litter and soil |
| A38 | *Alnus viridis* litter and soil |
| A39 | *Erica* litter and soil |
| A40 | *Erica* litter and soil |
| A41 | *Centaurea pseudophrygia* |
| A42 | *Centaurea pseudophrygia* |
| A43 | *Vaccinium* litter and soil |
| A44 | *Vaccinium* litter and soil |
| A45 | *Pinus cembra* litter |
| A46 | Rotten deadwood |
| A47 | Rotten deadwood |
| A48 | *Alnus viridis* litter and soil |
| A49 | *Alnus viridis* litter and soil |
| A50 | Soil beneath *Erica* |
| A51 | Soil beneath *Erica* |
| A52 | *Carlina acaulis* |
| A53 | Rotten deadwood |
| A54 | Rotten deadwood |
| A55 | Moss and soil |
| A56 | Cow feaces |
| A57 | Moss and soil |
| A58 | *Vaccinium myrtillus* fruits (blueberries) |
| A59 | Soil beneath *Vaccinium* |
| A60 | Sheep feaces |
| A61 | Mushroom indet. |
| A62 | Dry *Juniperus* fruits |
| A63 | Sheep feaces |
| A64 | Sheep feaces |
| A65 | Mushroom indet. |
| A66 | *Alchemilla vulgaris* agg. |
| A67 | Moss |
| A68 | *Alnus* leafes |
| A69 | *Taraxacum officinale* |
| A70 | Mushroom indet. |
| A71 | Fresh *Juniperus* fruits |
| A72 | Moss |
| A73 | Moss |
| A74 | Gall on *Rhododendron* |
| A75 | Sheep feaces |
| A76 | Deadwood |
| A77 | *Hieracium* sp. |
| A78 | *Taraxacum officinale* |
| A79 | Fresh *Juniperus* fruits |
| A80 | *Erica* fresh |
| A81 | *Pinus cembra* shoot |
| A82 | *Hieracium* cf. *lachenalii* |
| A83 | *Rhododendron* fresh with fruits |
| K 1 | *Pinus mugo* needles and shoots |
| K 2 | Moss and soil |
| K 3 | Deadwood |
| K 4 | Moss and grass with soil |
| K 5 | *Scabiosa columbaria* agg |
| K 6 | *Erica* litter and soil |
| K 7 | Cow feaces |
| K 8 | *Pinus mugo* litter |
| K 9 | Moss and soil |
| K 10 | *Erica* fresh and moss |
| K 11 | Moss beneath *Erica* |
| K 12 | *Scabiosa columbaria* agg |
| K 13 | *Pinus mugo* litter and soil |
| K 14 | *Pinus mugo* litter and soil |
| K 15 | *Salix appendiculata* fresh |
| K 16 | Lichens and soil |
| K 17 | Withered *Gentianella* sp. |
| K 18 | Mushroom indet. |
| K 19 | *Vaccinium vitis-idea* fruits (lingonberries) |
| K 20 | Cow feaces |
| K 21 | Cow feaces |
| K 22 | Erica fresh and soil |
| K 23 | Deadwood |
| K 24 | cf. *Leontodon* sp. |
| K 25 | *Juniperus* fruits |
| K 26 | *Fragaria* sp. fruits (wood strawberries) |
| K 27 | Cow feaces |
| K 28 | *Pinuicula* sp. |
| K 29 | Deadwood |
| K 30 | Deadwood |
| K 31 | *Vaccinium myrtillus* fruits (blueberries) |
| K 32 | *Vaccinium uliginosum* fruits (bog bilberries) |
| K 33 | *Vaccinium myrtillus* and *V. uliginosum* fruits (blueberries and bog bilberries) |
| K 34 | *Carlina acaulis* |
| K 35 | *Carlina acaulis* |
| K 36 | Deadwood |
| K 37 | *Rhododendron* fruits |
| K 38 | Moss |
| K 39 | *Pinus mugo* branches |
| K 40 | *Pinus mugo* litter |
| K 41 | *Leucanthemum* sp. |
| K 42 | cf. *Listera ovata* leafes |
| K 43 | *Hypericum maculatum* |
| K 44 | *Vaccinium vitis-idea* fruits (lingonberries) |
| K 45 | *Vaccinium uliginosum* fruits (bog bilberries) |
| K 46 | *Vaccinium myrtillus* fruits (blueberries) |
| K 47 | *Pinus mugo* litter and moss |
| K 48 | *Pinus mugo* litter and moss |
| K 49 | Mushroom indet. |
| K 50 | Mushroom indet. |
| K 51 | Mushroom indet. |
| K 52 | Mushroom indet. |
| K 53 | Mushroom indet. |
| K 54 | Mushroom indet. |
| K 55 | Mushroom indet. |
| K 56 | Mushroom indet. |
| K 57 | Mushroom indet. |
| K 58 | Mushroom indet. |
| K 59 | Mushroom indet. |
| K 60 | Mushroom indet. |
| K 61 | Mushroom indet. |
| K 62 | Mushroom indet. |
| K 63 | Mushroom indet. |
| K 64 | Mushroom indet. |
| K 65 | Mushroom indet. |
| K 66 | Mushroom indet. |
| K 67 | Mushroom indet. |
| K 68 | Mushroom indet. |
| K 69 | Mushroom indet. |
| K 70 | Mushroom indet. |
| K 71 | Mushroom indet. |
| K 72 | Mushroom indet. |
| K 73 | Mushroom indet. |
| K 74 | Mushroom indet. |
| K 75 | Mushroom indet. |
| K 76 | Mushroom indet. |
| K 77 | Mushroom indet. |
| K 78 | Mushroom indet. |
| K 79 | Mushroom indet. |
| K 80 | Mushroom indet. |
| K 81 | Mushroom indet. |
| K 82 | Mushroom indet. |
| K 83 | Mushroom indet. |
| K 84 | Mushroom indet. |
| K 85 | Mushroom indet. |
| K 86 | Mushroom indet. |
| K 87 | Mushroom indet. |
| K 88 | Mushroom indet. |
| K 89 | Mushroom indet. |
| K 90 | Mushroom indet. |
| K 91 | Mushroom indet. |
| K 92 | Mushroom indet. |
| K 93 | *Pinus mugo* needles and moss |
| K 94 | Lichens and soil |
| K 95 | *Salix appendiculata* |
| K 96 | *Fragaria* sp. fruits (wood strawberries) |
| P1 | Withered *Knautia* sp. |
| P2 | *Alnus viridis* fresh with fruits |
| P3 | *Alnus viridis* fresh with fruits |
| P4 | *Rumex alpestris* |
| P5 | *Rumex alpestris* |
| P6 | *Crepis* cf. *conyzifolia* |
| P7 | *Achillea millefolia* |
| P8 | *Achillea millefolia* |
| P9 | *Vaccinium myrtillus* fresh with fruits and roots |
| P10 | *Vaccinium myrtillus* fresh with fruits and roots |
| P11 | *Hypericum maculatum* |
| P12 | *Hypericum maculatum* |
| P13 | *Vaccinium myrtillus* fruits (blueberries) |
| P14 | *Juniperus* branch |
| P15 | *Rumex alpestris* |
| P16 | *Aconitum variegatum agg* |
| P17 | *Polygonatum verticillatum* |
| P18 | *Adenostyles alpina* leaf |
| P19 | *Rosa pendulina* fresh with fruits |
| P20 | *Leucantemum* sp. |
| P21 | Moss and soil |
| P22 | Plant indet. |
| P23 | *Carlina acaulis* |
| P24 | *Vaccinium myrtillus* fruits (blueberries) |
| P25 | *Rubus ideaus* fresh with fruits |
| P26 | *Gentianella* sp. |
| P27 | *Vaccinium uliginosum* fresh |
| P28 | *Aconitum variegatum* |
| P29 | *Alnus viridis* litter |
| P30 | *Alnus viridis* litter |
| P31 | *Chaerophyllum villarsii* |
| P32 | *Alnus viridis* litter |
| P33 | *Vaccinium vitis-idea* fresh with fruits |
| P34 | *Alnus viridis* litter |
| P35 | Plant indet. |
| P36 | *Vaccinium uliginosum* fruits (bog bilberries) |
| P37 | Plant indet. |
| P38 | *Vaccinium myrtillus* fruits (blueberries) |
| P39 | Deadwood and mushroom indet. |
| P40 | *Alnus viridis* fresh |
| P41 | *Solidago virgaurea* |
| P42 | *Alnus viridis* litter |
| P43 | Moss |
| P44 | *Chenopodium bonus-henricus* |
| P45 | *Galeopsis tetrahit* |
| P46 | *Galeopsis tetrahit* |
| P47 | *Galeopsis tetrahit* |
| P48 | *Alnus viridis* fresh with fruits |
| P49 | *Alnus viridis* litter |
| P50 | *Alnus viridis* litter |
| P51 | Deadwood |
| P52 | Deadwood and mushroom indet. |
| P53 | Moss and litter |
| P54 | *Pulsatilla alpina* |
| P55 | Mushroom indet. |
| P56 | *Hypericum maculatum* |
| P57 | *Alnus viridis* litter |
| P58 | *Rubus idaeus* branch and roots |
| P59 | *Solidago virgaurea* |

ID, substrate voucher including sample location information: A...Arztal, K...Kaserstattalm, P...Pfitscherjoch. Substrate, substrate type specification: substrates with the addition “fresh” included fresh leaves and branches; substrates with the addition “litter” included dead leaves and branches at early decaying stage; substrates with the addition “rotten” included dead leaves and branches at late decaying stage..
