# Supplementary material for: Oviposition Substrate of the Mountain Fly Drosophila nigrosparsa (Diptera: Drosophilidae)
Source: PLoS One. 2016 Oct 27;11(10):e0165743. doi: 10.1371/journal.pone.0165743 (PMC5082818; doi:10.1371/journal.pone.0165743)
Supplement: S2 Table — (DOC) [file pone.0165743.s002.doc]

**S2 Table. Experiment 2 −** General oviposition preference.

| Substrate | Replicate | Eggs | Adults |
| --- | --- | --- | --- |
| *Alnus* fresh | A | 0.0 | 0 |
|  | B | 0.0 | 0 |
|  | C | 0.0 | 0 |
|  | Mean ± SD | 0.0 ± 0.0 | 0.0 ± 0.0 |
| *Alnus* litter | A | 0.0 | 0 |
|  | B | 0.0 | 0 |
|  | C | 0.0 | 0 |
|  | Mean ± SD | 0.0 ± 0.0 | 0.0 ± 0.0 |
| *Alnus* rotten | A | 0.0 | 0 |
|  | B | 0.0 | 0 |
|  | C | 0.0 | 0 |
|  | Mean ± SD | 0.0 ± 0.0 | 0.0 ± 0.0 |
| Blueberries | A | 5.4 | 15 |
|  | B | 5.1 | 13 |
|  | C | 6.7 | 15 |
|  | Mean ± SD | 5.7 ± 0.8 | 14.3 ± 1.2 |
| Bog bilberries | A | 3.8 | 5 |
|  | B | 6.0 | 26 |
|  | C | 0.6 | 6 |
|  | Mean ± SD | 3.5 ± 2.7 | 12.3 ± 11.9 |
| Cow faeces | A | 0.0 | 0 |
|  | B | 0.0 | 0 |
|  | C | 0.0 | 0 |
|  | Mean ± SD | 0.0 ± 0.0 | 0.0 ± 0.0 |
| *Erica* fresh | A | 0.0 | 0 |
|  | B | 0.0 | 0 |
|  | C | 0.0 | 0 |
|  | Mean ± SD | 0.0 ± 0.0 | 0.0 ± 0.0 |
| Grass litter | A | 0.0 | 0 |
|  | B | 0.0 | 0 |
|  | C | 0.0 | 0 |
|  | Mean ± SD | 0.0 ± 0.0 | 0.0 ± 0.0 |
| *Juniperus* fresh | A | 0.0 | 0 |
|  | B | 0.0 | 0 |
|  | C | 0.0 | 0 |
|  | Mean ± SD | 0.0 ± 0.0 | 0.0 ± 0.0 |
| *Larix* fresh | A | 0.0 | 0 |
|  | B | 0.0 | 0 |
|  | C | 0.0 | 0 |
|  | Mean ± SD | 0.0 ± 0.0 | 0.0 ± 0.0 |
| *Larix* litter | A | 0.0 | 0 |
|  | B | 0.0 | 0 |
|  | C | 0.0 | 0 |
|  | Mean ± SD | 0.0 ± 0.0 | 0.0 ± 0.0 |
| *Larix* rotten | A | 0.0 | 0 |
|  | B | 0.0 | 0 |
|  | C | 0.0 | 0 |
|  | Mean ± SD | 0.0 ± 0.0 | 0.0 ± 0.0 |
| Lichens | A | 0.0 | 0 |
|  | B | 0.0 | 0 |
|  | C | 0.0 | 0 |
|  | Mean ± SD | 0.0 ± 0.0 | 0.0 ± 0.0 |
| Lingonberries | A | 0.0 | 0 |
|  | B | 0.0 | 0 |
|  | C | 0.0 | 0 |
|  | Mean ± SD | 0.0 ± 0.0 | 0.0 ± 0.0 |
| Moss | A | 0.0 | 0 |
|  | B | 0.0 | 0 |
|  | C | 0.0 | 0 |
|  | Mean ± SD | 0.0 ± 0.0 | 0.0 ± 0.0 |
| Mushrooms | A | 133.3 | 195 |
|  | B | 105.7 | 204 |
|  | C | 159.7 | 330 |
|  | Mean ± SD | 132.9 ± 27.0 | 243.0 ± 75.5 |
| *Pinus* fresh | A | 0.0 | 0 |
|  | B | 0.0 | 0 |
|  | C | 0.0 | 0 |
|  | Mean ± SD | 0.0 ± 0.0 | 0.0 ± 0.0 |
| *Pinus* litter | A | 0.0 | 0 |
|  | B | 0.0 | 0 |
|  | C | 0.0 | 0 |
|  | Mean ± SD | 0.0 ± 0.0 | 0.0 ± 0.0 |
| *Pinus* rotten | A | 0.0 | 0 |
|  | B | 0.0 | 0 |
|  | C | 0.0 | 0 |
|  | Mean ± SD | 0.0 ± 0.0 | 0.0 ± 0.0 |
| *Pinus* old cone | A | 0.0 | 0 |
|  | B | 0.0 | 0 |
|  | C | 0.0 | 0 |
|  | Mean ± SD | 0.0 ± 0.0 | 0.0 ± 0.0 |
| *Rhododendron* fresh | A | 0.0 | 0 |
|  | B | 0.0 | 0 |
|  | C | 0.0 | 0 |
|  | Mean ± SD | 0.0 ± 0.0 | 0.0 ± 0.0 |
| Sod | A | 0.0 | 0 |
|  | B | 0.0 | 0 |
|  | C | 0.0 | 0 |
|  | Mean ± SD | 0.0 ± 0.0 | 0.0 ± 0.0 |
| Soil | A | 0.0 | 0 |
|  | B | 0.0 | 0 |
|  | C | 0.0 | 0 |
|  | Mean ± SD | 0.0 ± 0.0 | 0.0 ± 0.0 |
| *Vaccinium* fresh | A | 0.0 | 0 |
|  | B | 0.0 | 0 |
|  | C | 0.0 | 0 |
|  | Mean ± SD | 0.0 ± 0.0 | 0.0 ± 0.0 |

Substrate, substrate type specification: For details about substrate nomenclature, see S1 Table. Eggs, number of eggs laid by 50 females/day when various substrates were available. SD, standard deviation. Adults, number of adults eclosed after placing the substrate on malt medium.
