# Supplementary material for: Oviposition Substrate of the Mountain Fly Drosophila nigrosparsa (Diptera: Drosophilidae)
Source: PLoS One. 2016 Oct 27;11(10):e0165743. doi: 10.1371/journal.pone.0165743 (PMC5082818; doi:10.1371/journal.pone.0165743)
Supplement: S3 Table — (DOC) [file pone.0165743.s003.doc]

**S3 Table. Experiment 3 −** Generalist vs. specialist.

| Substrate | Replicate | Eggs | Adults |
| --- | --- | --- | --- |
| Blueberries | A | 0.0 | 5 |
|  | B | 0.8 | - |
|  | C | 3.4 | - |
|  | Mean ± SD | 1.4 ± 1.8 | 5.0 ± 0.0 |
| Bog bilberries | A | 1.1 | - |
|  | B | 0.0 | 9 |
|  | C | 4.9 | - |
|  | Mean ± SD | 2.0 ± 2.6 | 9.0 ± 0.0 |
| *Inocybe terrigena* | A | 90.4 | - |
|  | B | 51.0 | - |
|  | C | 113.3 | - |
|  | Mean ± SD | 84.9 ± 31.5 | - |
| *Lactarius deterrimus* | A | 18.4 | - |
|  | B | 10.9 | - |
|  | C | 12.8 | - |
|  | Mean ± SD | 14.0 ± 3.9 | - |
| *Lactarius rufus* | A | 8.3 | - |
|  | B | 6.4 | - |
|  | C | 33.0 | - |
|  | Mean ± SD | 15.9 ± 14.9 | - |
| *Lycoperdon* sp. & | A | 18.8 | - |
| *Bovista* sp. | B | 25.1 | - |
|  | C | 77.3 | - |
|  | Mean ± SD | 40.4 ± 32.1 | - |
| *Russula sp.* | A | 38.3 | - |
|  | B | 82.1 | - |
|  | C | 85.5 | - |
|  | Mean ± SD | 68.6 ± 26.4 | - |
| *Suillus grannulatus* | A | 8.6 | - |
|  | B | 3.4 | - |
|  | C | 1.5 | - |
|  | Mean ± SD | 4.5 ± 3.7 | - |
| *Tricholoma vaccinum* | A | 0.0 | 0 |
|  | B | 7.5 | - |
|  | C | 0.0 | 0 |
|  | Mean ± SD | 2.5 ± 4.3 | 0.0 ± 0.0 |

Substrate, substrate type specification. Eggs, number of eggs laid by 50 females/day when different kinds of favoured substrates were available. SD, standard deviation. Adults, the number of adults eclosed after placing the substrate on malt medium. - substrate was not placed on malt medium because eggs were found in Experiment 3.
