# Supplementary material for: Oviposition Substrate of the Mountain Fly Drosophila nigrosparsa (Diptera: Drosophilidae)
Source: PLoS One. 2016 Oct 27;11(10):e0165743. doi: 10.1371/journal.pone.0165743 (PMC5082818; doi:10.1371/journal.pone.0165743)
Supplement: S4 Table — (DOC) [file pone.0165743.s004.doc]

**S4 Table. Experiment 4 − Opportunistic ovipositing**.

| Substrate | Replicate | Eggs | Adults |
| --- | --- | --- | --- |
| *Alnus* fresh | A | 15.4 | - |
|  | B | 0.0 | 3 |
|  | C | 0.4 | - |
|  | Mean ± SD | 5.3 ± 8.8 | 3.0 ± 0.0 |
| *Alnus* litter | A | 2.6 | - |
|  | B | 7.1 | - |
|  | C | 0.8 | - |
|  | Mean ± SD | 3.5 ± 3.3 | - |
| *Alnus* rotten | A | 0.0 | 0 |
|  | B | 0.0 | 0 |
|  | C | 0.0 | 0 |
|  | Mean ± SD | 0.0 ± 0.0 | 0.0 ± 0.0 |
| Cow faeces | A | 0.0 | 0 |
|  | B | 0.0 | 3 |
|  | C | 0.0 | 0 |
|  | Mean ± SD | 0.0 ± 0.0 | 1.0 ± 1.7 |
| Lingonberries | A | 0.0 | 2 |
|  | B | 0.0 | 0 |
|  | C | 0.0 | 0 |
|  | Mean ± SD | 0.0 ± 0.0 | 0.67 ± 1.2 |
| Moss | A | 0.0 | 0 |
|  | B | 0.0 | 0 |
|  | C | 0.0 | 0 |
|  | Mean ± SD | 0.0 ± 0.0 | 0.0 ± 0.0 |
| *Pinus* fresh | A | 0.0 | 0 |
|  | B | 2.6 | - |
|  | C | 0.0 | 7 |
|  | Mean ± SD | 0.9 ± 1.5 | 3.5 ± 5.0 |
| *Pinus* litter | A | 0.0 | 0 |
|  | B | 0.0 | 0 |
|  | C | 0.0 | 0 |
|  | Mean ± SD | 0.0 ± 0.0 | 0.0 ± 0.0 |
| *Pinus* rotten | A | 0.0 | 13 |
|  | B | 0.0 | 0 |
|  | C | 0.0 | 0 |
|  | Mean ± SD | 0.0 ± 0.0 | 4.3 ± 7.5 |

Substrate, substrate type specification: For details about substrate nomenclature, see S1 Table. Eggs, number of eggs laid by 50 females/day when no favoured substrates were available. SD, standard deviation. Adults, the number of adults eclosed after placing the substrate on malt medium. - substrate was not placed on malt medium because eggs were found in Experiment 4.
