# Supplementary material for: Oviposition Substrate of the Mountain Fly Drosophila nigrosparsa (Diptera: Drosophilidae)
Source: PLoS One. 2016 Oct 27;11(10):e0165743. doi: 10.1371/journal.pone.0165743 (PMC5082818; doi:10.1371/journal.pone.0165743)
Supplement: S5 Table — (DOC) [file pone.0165743.s005.doc]

| ID | Substrate | Experiment | Replicate | % ILP |
| --- | --- | --- | --- | --- |
| s44 | Blueberries | 3 | A | 100.0 |
| s45 | Blueberries | 3 | A | 100.0 |
| s46 | Blueberries | 3 | A | 100.0 |
| s47 | Blueberries | 3 | A | 100.0 |
| s48 | Blueberries | 3 | A | 100.0 |
| s35 | Bog bilberries | 3 | B | 100.0 |
| s36 | Bog bilberries | 3 | B | 100.0 |
| s37 | Bog bilberries | 3 | B | 100.0 |
| s38 | Bog bilberries | 3 | B | 100.0 |
| s39 | Bog bilberries | 3 | B | 100.0 |
| s40 | Bog bilberries | 3 | B | 100.0 |
| s41 | Bog bilberries | 3 | B | 100.0 |
| s42 | Bog bilberries | 3 | B | 100.0 |
| s43 | Bog bilberries | 3 | B | 100.0 |
| s33 | *Alnus* fresh | 4 | B | 100.0 |
| s34 | *Alnus* fresh | 4 | B | 100.0 |
| s49 | Cow faeces | 4 | B | 100.0 |
| s50 | Cow faeces | 4 | B | 100.0 |
| s51 | Cow faeces | 4 | B | 100.0 |
| s52 | Lingonberries | 4 | A | 100.0 |
| s53 | Lingonberries | 4 | A | 97.4 |
| s54 | *Pinus* fresh | 4 | C | 100.0 |
| s55 | *Pinus* fresh | 4 | C | 100.0 |
| s56 | *Pinus* fresh | 4 | C | 100.0 |
| s57 | *Pinus* fresh | 4 | C | 100.0 |
| s58 | *Pinus* fresh | 4 | C | 100.0 |
| s59 | *Pinus* fresh | 4 | C | 100.0 |
| s60 | *Pinus* fresh | 4 | C | 100.0 |
| s61 | *Pinus* rotten | 4 | A | 100.0 |
| s62 | *Pinus* rotten | 4 | A | 100.0 |
| s63 | *Pinus* rotten | 4 | A | 100.0 |
| s64 | *Pinus* rotten | 4 | A | 100.0 |
| s65 | *Pinus* rotten | 4 | A | 100.0 |
| s66 | *Pinus* rotten | 4 | A | 100.0 |
| s67 | *Pinus* rotten | 4 | A | 100.0 |
| s68 | *Pinus* rotten | 4 | A | 100.0 |
| s69 | *Pinus* rotten | 4 | A | 100.0 |
| s70 | *Pinus* rotten | 4 | A | 100.0 |
| s71 | *Pinus* rotten | 4 | A | 100.0 |
| s72 | *Pinus* rotten | 4 | A | 100.0 |

ID, specimen voucher. Substrate, substrate type specification: For details about the substrate nomenclature, see S1 Table. Experiment, 3...Experiment 3 − Generalist vs. specialist, 4...Experiment 4 − Opportunistic breeding. Replicate, replicate cage within each experiment. % ILP, the probability that the genotype is part of the inbred laboratory population in percent.
