# Supplementary material for: Oviposition Substrate of the Mountain Fly Drosophila nigrosparsa (Diptera: Drosophilidae)
Source: PLoS One. 2016 Oct 27;11(10):e0165743. doi: 10.1371/journal.pone.0165743 (PMC5082818; doi:10.1371/journal.pone.0165743)
Supplement: S6 Table — (DOC) [file pone.0165743.s006.doc]

| ID | Substrate | Individuals | Species | Family | Order |
| --- | --- | --- | --- | --- | --- |
| A36 | Moss | 1 | *Otiorhynchus coecus* | Curculionidae | Coleoptera |
| A75 | Sheep faeces | 1 | *Cercyon* cf. *impressus* | Hydrophilidae | Coleoptera |
| K20 | Cow faeces | 1 | *Acrotrichis* sp. | Ptilidae | Coleoptera |
| A75 | Sheep faeces | 6 | *Acrotrichis* sp. | Ptilidae | Coleoptera |
| A56 | Cow faeces | 1 | - | Staphylinidae | Coleoptera |
| A75 | Sheep faeces | 1 | - | Staphylinidae | Coleoptera |
| P49 | *Alnus* litter | 1 | - | Cecidomyiidae | Diptera |
| A49 | *Alnus viridis* litter and soil | 1 | - | Cecidomyiidae | Diptera |
| K27 | Cow faeces | 3 | - | Cecidomyiidae | Diptera |
| K48 | *Pinus mugo* litter and moss | 3 | - | Cecidomyiidae | Diptera |
| A60 | Sheep faeces | 41 | - | Cecidomyiidae | Diptera |
| A63 | Sheep faeces | 1 | - | Cecidomyiidae | Diptera |
| A75 | Sheep faeces | 1 | - | Cecidomyiidae | Diptera |
| A56 | Cow faeces | 5 | - | Chironomidae | Diptera |
| K20 | Cow faeces | 94 | - | Chironomidae | Diptera |
| K21 | Cow faeces | 4 | - | Chironomidae | Diptera |
| K27 | Cow faeces | 2 | - | Chironomidae | Diptera |
| K7 | Cow faeces | 90 | - | Chironomidae | Diptera |
| K73 | Mushroom indet. | 2 | - | Chironomidae | Diptera |
| A60 | Sheep faeces | 3 | - | Chironomidae | Diptera |
| A63 | Sheep faeces | 11 | - | Chironomidae | Diptera |
| A75 | Sheep faeces | 56 | - | Chironomidae | Diptera |
| A32 | Mushroom indet. | 2 | *Drosophila transversa* | Drosophilidae | Diptera |
| K50 | Mushroom indet. | 24 | *Drosophila transversa* | Drosophilidae | Diptera |
| K53 | Mushroom indet. | 2 | *Drosophila transversa* | Drosophilidae | Diptera |
| K54 | Mushroom indet. | 7 | *Drosophila transversa* | Drosophilidae | Diptera |
| K61 | Mushroom indet. | 91 | *Drosophila transversa* | Drosophilidae | Diptera |
| K70 | Mushroom indet. | 1 | *Drosophila transversa* | Drosophilidae | Diptera |
| K71 | Mushroom indet. | 13 | *Drosophila transversa* | Drosophilidae | Diptera |
| K89 | Mushroom indet. | 12 | *Drosophila transversa* | Drosophilidae | Diptera |
| K20 | Cow faeces | 2 | - | Empididae | Diptera |
| K7 | Mushroom indet. | 1 | - | Limonidae | Diptera |
| K83 | Mushroom indet. | 1 | - | Muscidae | Diptera |
| K55 | Mushroom indet. | 2 | - | Mycetophilidae | Diptera |
| A30 | *Alnus viridis* litter | 1 | - | Phoridae | Diptera |
| A56 | Cow faeces | 3 | - | Psychodidae | Diptera |
| K7 | Cow faeces | 1 | - | Psychodidae | Diptera |
| K59 | Mushroom indet. | 16 | - | Psychodidae | Diptera |
| A30 | *Alnus viridis* litter | 4 | - | Sciaridae | Diptera |
| P50 | *Alnus viridis* litter | 3 | - | Sciaridae | Diptera |
| P52 | Dead wood & mushroom indet. | 1 | - | Sciaridae | Diptera |
| K73 | Mushroom indet. | 1 | - | Sciaridae | Diptera |
| A60 | Sheep faeces | 38 | - | Sciaridae | Diptera |
| A75 | Sheep faeces | 3 | - | Sciaridae | Diptera |
| A56 | Cow faeces | 1 | - | Sepsidae | Diptera |
| K7 | Cow faeces | 1 | - | Sepsidae | Diptera |
| A56 | Cow faeces | 2 | - | Sphaeroceidae | Diptera |
| K20 | Cow faeces | 19 | - | Sphaeroceidae | Diptera |
| K21 | Cow faeces | 11 | - | Sphaeroceidae | Diptera |
| A75 | Sheep faeces | 11 | - | Sphaeroceidae | Diptera |
| K24 | cf. *Leontodon* sp. | 16 | - | Tephritidae | Diptera |
| A82 | *Hieracium* cf. *lachenalii* | 1 | - | Tephritidae | Diptera |
| A77 | *Hieracium* sp. | 3 | - | Tephritidae | Diptera |
| P37 | Plant indet. | 1 | - | Tephritidae | Diptera |
| K54 | Mushroom indet. | 1 | - | Tipulidae | Diptera |
| A22 | *Cirsium heterophyllum* | 18 | - | - | Hemiptera |
| A6 | Moss and soil | 1 | *Kleidocerys resedae* | Lygaeidae | Hemiptera |
| A16 | *Rhododendron* branch and leafes | 1 | *Kleidocerys resedae* | Lygaeidae | Hemiptera |
| A56 | Cow faeces | 1 | - | cf. Figitidae | Hymenoptera |
| A1 | Lichens on *Picea abies* branch | 1 | - | cf. Pteromalidae | Hymenoptera |
| K17 | Withered *Gentianella* sp. | 40 | - | - | Thysanoptera |
| K54 | Mushroom indet. | 1 | - | - | Thysanoptera |

ID, substrate voucher including sample location information: A...Arztal, K...Kaserstattalm, P...Pfitscherjoch. Substrate, substrate type specification: For details about substrate nomenclature, see S1 Table. Individuals, number of individuals eclosed. - indicate missing identification.
